# A Novel Mouse Model for Venous High-Load Remodeling Induced by Unilateral Jugular Vein Transection

**DOI:** 10.3390/jcdd13090452

**Published:** 2026-09-10

**Authors:** Ke Hu, Shiwen Yu, Peng Tang, Wangxuan Lv, Junfei Zhu, Junli Zhuang, Shunchang Zhou, Hongping Deng

**Affiliations:** 1 Department of Vascular Surgery, Renmin Hospital of Wuhan University, Wuhan 430060, China; rm004312@whu.edu.cn (K.H.); tangpeng1507@whu.edu.cn (P.T.); rm002999@whu.edu.cn (J.Z.); 2 Department of Vascular Surgery, Union Hospital, Tongji Medical College, Huazhong University of Science and Technology, Wuhan 430022, China; d202672663@hust.edu.cn (S.Y.); m202476390@hust.edu.cn (W.L.); m202576357@hust.edu.cn (J.Z.); 3 Center of Experimental Animals, Huazhong University of Science and Technology, Wuhan 430074, China

**Keywords:** venous high load, mouse model, jugular vein transection, smooth muscle phenotypic switching, extracellular matrix remodeling, vascular remodeling

## Abstract

Chronic venous disease (CVD) is a prevalent peripheral vascular disorder characterized by vascular remodeling driven by hemodynamic overload, yet available animal models are limited by high surgical difficulty and unstable induction efficiency. Here, we developed a novel mouse venous high-load remodeling model by selectively ablating the unilateral cervical venous drainage system. After 4 weeks, compensatory contralateral jugular veins were assessed via ultrasonography, histology, Western blot, and bulk RNA sequencing. The model displayed marked luminal dilation, wall hypertrophy, and excessive collagen deposition, along with downregulated smooth muscle contractile markers (α-SMA, SM22α) and upregulated adhesion molecule ICAM-1. Transcriptomic profiling identified 252 differentially expressed genes, predominantly enriched in extracellular matrix organization, angiogenesis, and complement-coagulation cascades, suggesting potential pathways involved in the venous remodeling process. This model reliably recapitulates the pathological features associated with hemodynamic overload-induced venous remodeling, offering a valuable tool for investigating CVD pathogenesis and intervention targets.

## 1. Introduction

Chronic venous disease (CVD) is a prevalent peripheral vascular disorder in clinical practice, whose pathological core lies in venous hypertension-induced structural remodeling and functional dysfunction of the vascular wall [1,2]. Sustained venous hypertension leads to luminal dilation, wall thickening, and excessive deposition of extracellular matrix, accompanied by phenotypic switching of vascular smooth muscle cells (VSMCs) and endothelial inflammatory responses [3]. These pathological alterations ultimately contribute to the development of varicose veins, cutaneous nutritional impairment, and even venous ulceration.

Currently, available animal models for investigating venous hypertension-triggered vascular remodeling mainly include the rat femoral arteriovenous fistula model and the mouse hindlimb vein ligation model. However, these models are limited by drawbacks such as high surgical complexity, low efficiency of venous hypertension induction, and uncontrollable collateral blood flow pathways [4]. Accordingly, establishing a mouse model with simple operability, high reproducibility, and accurate simulation of the hemodynamic characteristics of human venous hypertension is of great significance for further elucidating the molecular mechanisms underlying venous hypertension-associated vascular lesions.

In the present study, we successfully established a novel mouse venous high-load (VH) remodeling model by selectively disrupting the unilateral cervical venous drainage system in mice. Four weeks after surgery, the compensatory left external jugular vein exhibited remarkable morphological remodeling, characterized by luminal dilation, wall thickening, and massive collagen fiber deposition (evidenced by a significant increase in the positive area of Masson’s trichrome staining). Meanwhile, the model showed downregulated expression of classic VSMC contractile phenotypic markers, namely α-smooth muscle actin (α-SMA) and SM22α, which was consistent with the contractile-to-synthetic phenotypic transition of VSMCs observed in clinical varicose vein specimens in previous studies [5]. Further investigations revealed that VH also significantly upregulated the expression of endothelial adhesion molecules intercellular cell adhesion molecule-1 (ICAM-1) and vascular cell adhesion molecule-1 (VCAM-1), indicating that endothelial inflammatory responses were effectively activated in this model [6].

Using this novel VH remodeling model, this study aims to systematically evaluate the molecular features of VH-induced vascular structural remodeling, VSMC phenotypic switching, and endothelial inflammatory responses, so as to provide a reliable experimental platform for further exploring the pathogenesis and potential intervention targets of VH-related vascular diseases.

## 2. Materials and Methods

### 2.1. Experimental Animals and Grouping

Male specific pathogen-free (SPF) C57BL/6J mice (8 weeks old, 22–25 g body weight) were purchased from Beijing Vital River Laboratory Animal Technology Co., Ltd. (Beijing, China) [production license no. SCXK (Jing) 2021-0006]. All animals were housed in a temperature- and humidity-controlled barrier environment (22 ± 2 °C, 50% ± 10% relative humidity, 12-h light/dark cycle) with ad libitum access to food and water. After 1 week of acclimatization, mice were stratified by body weight and then randomly assigned to the VH group and the sham-operated (Sham) group using a computer-generated random number table (Excel RAND function). A total of 26 mice were included, with 13 animals per group. Five mice per group were allocated for ultrasonographic, histological, and immunofluorescence analyses; 5 mice per group were used for Western blotting (with 6 technical replicates per sample); and 3 mice per group were used for RNA sequencing. No formal power calculation was performed prior to the experiment; the sample size was determined empirically based on our previous experience and standard practices in similar mouse vascular remodeling studies, which are sufficient to detect biologically meaningful differences.

This study was approved by the Institutional Animal Care and Use Committee of Huazhong University of Science and Technology (approval no. IACUC-4957). All experimental procedures were performed in compliance with the Guide for the Care and Use of Laboratory Animals and the ARRIVE guidelines.

### 2.2. Establishment of the Venous Hypertension Mouse Model

Mice were anesthetized via intraperitoneal injection of tribromoethanol (250 mg/kg) and fixed in the supine position on a thermostatic operating table. After neck shaving and disinfection, a longitudinal midline cervical incision was made. Under a stereomicroscope (SZ61, Olympus, Tokyo, Japan), the right external jugular vein and internal jugular vein were exposed via blunt dissection. For the VH group, the proximal and distal ends of the right external jugular vein trunk, as well as the ipsilateral internal jugular vein, were ligated with 8-0 silk suture and transected to completely block right cervical venous drainage and induce compensatory VH in the contralateral left external jugular vein. For the Sham group, vessels were only dissected without ligation or transection. The incision was sutured with 6-0 nylon suture and disinfected postoperatively. Intramuscular penicillin (40,000 U/mouse) was administered for 3 consecutive days to prevent infection, and mice were housed for 4 weeks before sample collection.

### 2.3. Ultrasonographic Assessment and Sample Collection

At 4 weeks post-surgery, mice were anesthetized with isoflurane inhalation, and the left external jugular vein was examined using a high-frequency small-animal ultrasound system (MYLAB^TM^ X5 VET, Esaote, Genova, Italy). The minimum luminal diameter was measured in B-mode and averaged over three consecutive cardiac cycles; blood flow direction and filling status were observed via color Doppler mode to verify compensatory venous return. All ultrasound measurements were performed by an investigator blinded to the group allocation.

After ultrasonography, mice were euthanized, and the left external jugular vein was fully dissected and rinsed of residual blood with pre-cooled PBS. The maximum outer circumference of the vessel was measured under a stereomicroscope. Each vessel was divided into two segments: one was fixed in 4% paraformaldehyde and embedded in paraffin for histological staining, and the other was snap-frozen in liquid nitrogen and stored at −80 °C for protein extraction and RNA sequencing.

### 2.4. Histological and Immunofluorescence Staining

Paraffin-embedded tissues were sectioned serially at 4 μm thickness and processed for the following staining after deparaffinization and rehydration:

Hematoxylin–eosin (HE) staining: Staining was performed using an HE staining kit (catalog no. G1120, Solarbio, Beijing, China) to observe the layered structure of the venous wall, medial smooth muscle arrangement, and intimal integrity.

Masson’s trichrome staining: Staining was conducted following the manufacturer’s protocol (catalog no. G1340, Solarbio, China), with collagen fibers stained blue and muscle fibers stained red. Five non-overlapping fields (×200 magnification) were randomly selected per section, and the percentage of collagen-positive area relative to the total venous wall area was calculated using ImageJ (v1.54f) software.

ICAM-1 immunofluorescence staining: Antigen retrieval was performed by microwave heating in citrate buffer (pH 6.0, catalog no. C1032, Solarbio, China) for 10 min. After washing with PBS, sections were blocked with 5% BSA for 30 min at room temperature, then incubated with anti-ICAM-1 primary antibody (1:100, catalog no. 10020-1-AP, Proteintech, Wuhan, China) overnight at 4 °C. After washing, sections were incubated with Alexa Fluor 488 goat anti-rabbit secondary antibody (1:500, catalog no. A-11008, Thermo Fisher Scientific, Waltham, MA, USA) for 1 h at room temperature in the dark. Nuclei were counterstained with DAPI-containing anti-fade mounting medium (catalog no. P0131, Beyotime, Shanghai, China). Images were captured with a laser confocal microscope (LSM880, Zeiss, Oberkochen, Germany). For quantitative analysis, three random fields of view were captured and averaged per tissue section as technical replicates, with *n* = 5 independent mice per group as biological replicates. Mean fluorescence intensity was quantified using ImageJ by an investigator blinded to the experimental groups.

For Collagen I (COL1), Collagen III (COL3), and Fibronectin (FN) immunofluorescence staining, sections were subjected to antigen retrieval in citrate buffer (pH 6.0) by microwave heating for 10 min. After blocking with 5% BSA for 30 min at room temperature, sections were incubated with primary antibodies against COL1 (1:200, catalog no. 14695-1-AP, Proteintech, China), COL3 (1:200, catalog no. 22734-1-AP, Proteintech, China), or FN (1:200, catalog no. 15613-1-AP, Proteintech, China) overnight at 4 °C. After washing, sections were incubated with Alexa Fluor 488-conjugated goat anti-rabbit secondary antibody (1:500, catalog no. A-11008, Thermo Fisher Scientific, USA) for 1 h at room temperature in the dark. Nuclei were counterstained with DAPI-containing mounting medium. Images were captured with a laser confocal microscope. For quantitative analysis, three random fields of view were captured and averaged per tissue section as technical replicates, with *n* = 5 independent mice per group as biological replicates. Mean fluorescence intensity was quantified using ImageJ by an investigator blinded to the experimental groups.

For double immunofluorescence staining of ICAM-1 and PECAM-1 (CD31), sections were incubated with a mixture of anti-ICAM-1 (1:100, catalog no. 10020-1-AP, Proteintech, China) and anti-PECAM-1 (1:100, catalog no. 28083-1-AP, Proteintech, China) primary antibodies overnight at 4 °C, followed by incubation with Alexa Fluor 488-conjugated goat anti-rabbit (1:500) and Alexa Fluor 594-conjugated goat anti-mouse (1:500, catalog no. A-11005, Thermo Fisher Scientific, USA) secondary antibodies for 1 h at room temperature in the dark. Images were captured with a laser confocal microscope. For quantitative analysis, three random fields of view were captured and averaged per tissue section as technical replicates, with *n* = 5 independent mice per group as biological replicates. Mean fluorescence intensity was quantified using ImageJ by an investigator blinded to the experimental groups.

### 2.5. Western Blot Analysis

Frozen venous tissues were homogenized on ice in RIPA lysis buffer (catalog no. P0013B, Beyotime, China) supplemented with 1 mmol/L PMSF (catalog no. ST506, Beyotime, China) at a ratio of 30 mg tissue per 300 μL buffer. After centrifugation at 12,000 rpm for 15 min at 4 °C, the supernatant was collected as total protein extract. Protein concentration was determined using a BCA protein assay kit (catalog no. P0010, Beyotime, China).

Equal amounts of protein (30 μg per lane) were separated via 10% SDS-PAGE and transferred onto 0.45 μm PVDF membranes (catalog no. IPVH00010, Millipore, Billerica, MA, USA) via wet transfer. After blocking with 5% skim milk for 1 h at room temperature, membranes were incubated with primary antibodies overnight at 4 °C. Following TBST washes, membranes were incubated with HRP-conjugated goat anti-rabbit secondary antibody (1:5000, catalog no. RGAR001, Proteintech, China) for 1 h at room temperature. Protein bands were visualized using ECL chemiluminescent substrate (catalog no. WBKLS0500, Millipore, USA) with a ChemiDoc XRS+ imaging system (Bio-Rad, Hercules, CA, USA). Band gray values were analyzed with Image Lab software (v6.1) by an investigator blinded to the group allocation, and relative protein expression was calculated by normalizing target protein signals to the internal reference β-Tubulin, with the Sham group set as 1.0.

Primary antibodies for Western blot (all from Proteintech, China): anti-α-SMA (1:1000, 14395-1-AP), anti-SM22α (1:1000, 10493-1-AP), anti-ICAM-1 (1:1000, 10020-1-AP), anti-VCAM-1 (1:1000, 11444-1-AP), anti-β-Tubulin (1:2000, 10094-1-AP).

### 2.6. RNA Sequencing and Bioinformatic Analysis

Total RNA was extracted from left jugular vein tissues (3 biological replicates per group) using TRIzol reagent (catalog no. 15596026, Invitrogen, Carlsbad, CA, USA). RNA integrity and purity were assessed with an Agilent 2100 Bioanalyzer (Agilent Technologies, Santa Clara, CA, USA), and samples with RNA integrity number (RIN) ≥ 7.0 were used for library construction. cDNA libraries were prepared with the NEBNext Ultra II RNA Library Prep Kit (NEB, Ipswich, MA, USA) and subjected to paired-end 150 bp high-throughput sequencing on the Illumina NovaSeq 6000 platform.

After quality control and adapter trimming, clean reads were aligned to the mouse mm10 (GRCm38) reference genome using STAR software (v2.7.10a), and differentially expressed genes (DEGs) were identified with DESeq2 (v1.34.0; threshold: |log_2_fold change| > 1, adjusted *p*-value < 0.05). GO functional enrichment and KEGG pathway enrichment analyses were performed using the clusterProfiler (v4.2.2) R package. Gene set enrichment analysis (GSEA) was conducted with the fgsea (v1.20.0) package based on the MSigDB Hallmark gene set (threshold: |NES| > 1, adjusted *p*-value < 0.05). A protein–protein interaction (PPI) network was constructed using the STRING v11.5 database (confidence score > 0.4) and visualized with Cytoscape 3.9.1 software. All bioinformatic analyses were performed in a blinded manner with respect to group identity.

### 2.7. Statistical Analysis

No a priori power analysis was conducted for this exploratory animal study. The sample sizes were empirically determined based on our laboratory’s experience and established protocols for murine vascular remodeling models, which are adequate to detect consistent and biologically meaningful intergroup differences. All quantitative data are presented as mean ± standard error of the mean (SEM), and mean differences with 95% confidence intervals (95% CIs) for key comparative outcomes are provided in Supplementary Table S1. All statistical analyses were performed using GraphPad Prism 9.0 software. Prior to parametric testing, the Shapiro–Wilk test and F-test were used to verify data normality and homogeneity of variances, respectively, and no violations of parametric assumptions were detected. Independent two-tailed Student’s *t*-tests were applied for two-group comparisons, and a *p* value < 0.05 was considered statistically significant.

## 3. Results

### 3.1. Establishment of a Novel Mouse Model of Venous Hypertension

To further investigate the underlying molecular mechanisms of vascular remodeling induced by VH, we developed a novel mouse VH remodeling model in the present study. The core strategy of this model is to selectively disrupt the unilateral (right) cervical venous drainage system in mice, forcing compensatory blood return through the contralateral (left) external jugular vein.

**Figure 1:**
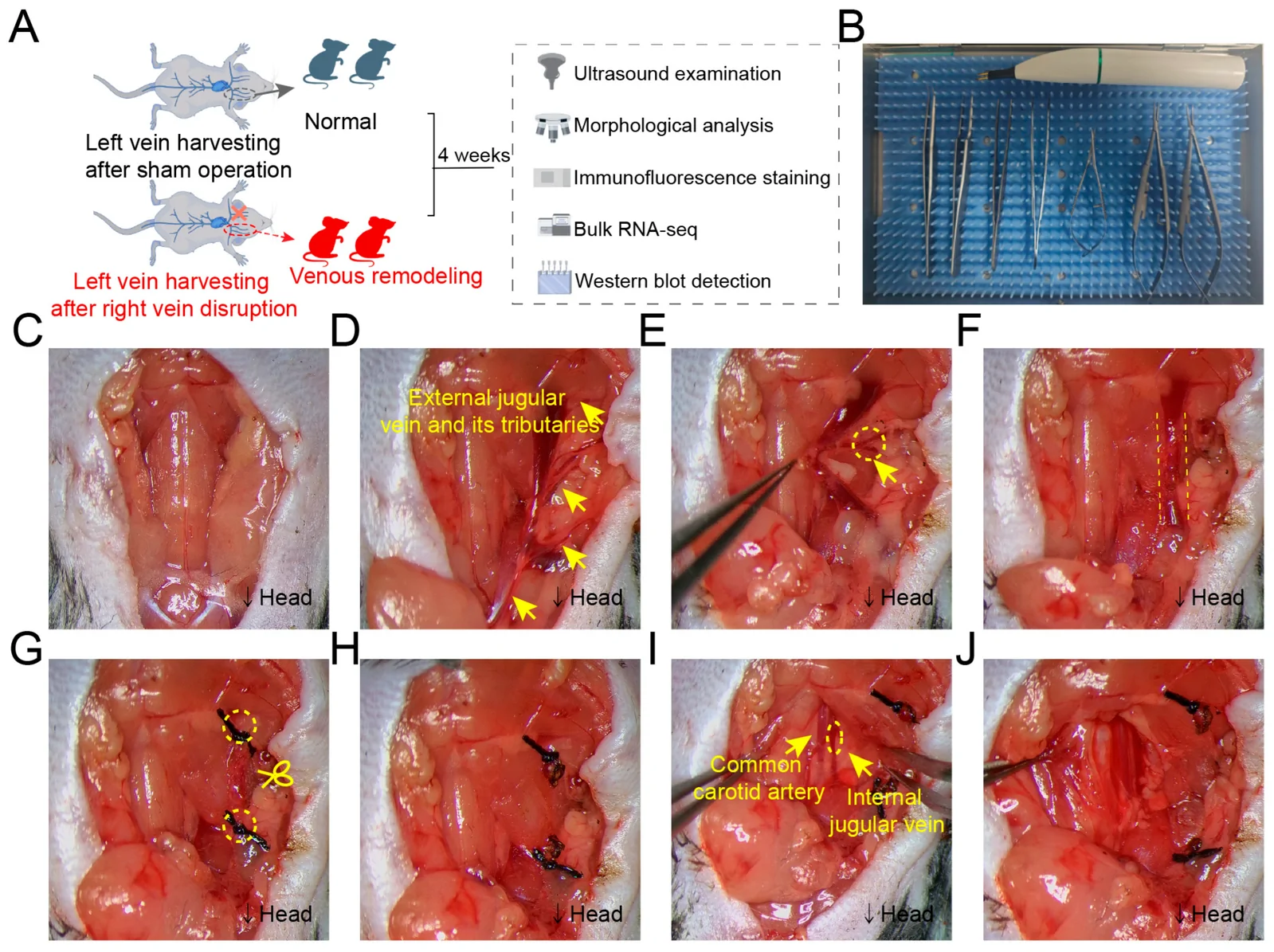
Establishment and validation of the mouse VH remodeling model induced by unilateral jugular vein ligation and transection. (**A**) Schematic diagram of the unilateral jugular vein ligation and transection procedure. (**B**) Intraoperative photograph showing the surgical field. (**C**) Gross view of isolated contralateral venous tissue and surrounding fascia. (**D**) Exposure of the superficial branch of the right external jugular vein. (**E**) Exposure of the deep branch of the right external jugular vein. (**F**) Blunt dissection of the right external jugular vein. (**G**) Ligation of the trunk of the right external jugular vein. (**H**) Transection of the ligated right external jugular vein. (**I**) Exposure of the right internal jugular vein. (**J**) Transection of the right internal jugular vein.

At 4 weeks postoperatively, compensatory veins from the experimental group and normal veins from age-matched sham-operated mice were harvested for ultrasonographic assessment, morphological measurement, immunofluorescence staining, bulk RNA sequencing, and Western blot analysis (Figure 1A). All surgical procedures were performed under a stereomicroscope with microsurgical instruments (Figure 1B).

The detailed surgical workflow is described as follows. First, a longitudinal skin incision was made along the anterior cervical midline. Under stereomicroscopy, the external jugular vein and its superficial and deep tributaries were carefully dissected (Figure 1C–E). After adequate mobilization of the tributaries, the main trunk of the external jugular vein was fully exposed (Figure 1F), followed by ligation and transection at both its proximal and distal ends (Figure 1G,H). Subsequently, the ipsilateral internal jugular vein was exposed and transected (Figure 1I,J). The above procedures achieved complete occlusion of the right cervical venous system. At the end of surgery, subcutaneous tissues were repositioned, the skin incision was sutured, and local disinfection was performed.

### 3.2. Morphological Remodeling and Fibrotic Changes in the Left External Jugular Vein Induced by Venous Hypertension

**Figure 2:**
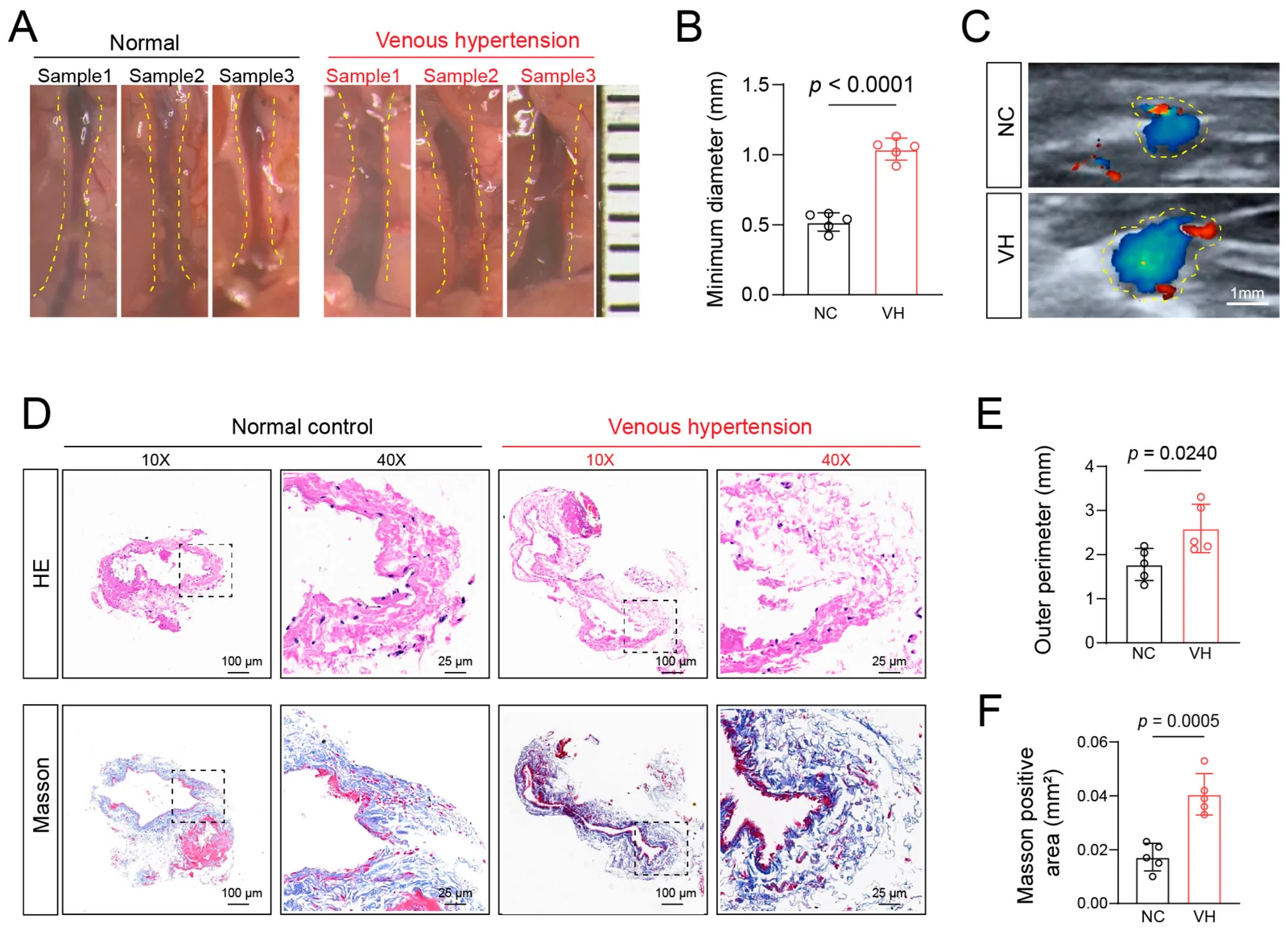
Histomorphological observation and special staining validation of venous tissues in sham (NC) and VH groups. (**A**) Gross photographs of cervical veins from NC and VH mice. (**B**) Comparison of venous tissue diameter between NC and VH groups (*p* < 0.0001, n = 5). (**C**) Representative cross-sectional ultrasound images of contralateral veins. (**D**) HE staining (×10 and ×40) showing venous wall structure, smooth muscle thickness, luminal morphology, and smooth muscle layer arrangement. Masson’s trichrome staining (×10 and ×40) showing collagen fiber distribution (blue) and extracellular matrix deposition. (**E**) Quantitative analysis of venous luminal perimeter (*p* = 0.0240, n = 5). (**F**) Quantitative analysis of collagen area fraction by Masson’s trichrome staining (*p* = 0.0005, n = 5). Data are presented as mean ± SEM.

At 4 weeks post-surgery, gross anatomical observation revealed that the left external jugular vein in the VH group exhibited obvious luminal dilation and tortuous course compared with the normal control (NC) group (Figure 2A). Ultrasonographic results showed that the minimum venous diameter in the VH group was significantly increased relative to the NC group (Figure 2B); meanwhile, the venous outer circumference in the VH group was also markedly larger than that in the NC group (Figure 2C).

For histological evaluation, HE staining demonstrated that the venous wall in the NC group presented an intact structure, with a smooth intimal surface and a regularly arranged medial smooth muscle layer of uniform thickness. In contrast, the venous wall in the VH group was significantly thickened, accompanied by apparent hyperplasia and disorganized arrangement of medial smooth muscle cells, and disruption of endothelial cell continuity was observed beneath the intima in some regions (Figure 2D). Even in the absence of intravascular blood tension, the VH group still displayed an enlarged vascular lumen (Figure 2E).

Masson’s trichrome staining further indicated that collagen fibers (stained blue) in the NC group were mainly confined to the adventitia, with low abundance and sparse distribution. In the VH group, however, extensive blue-positive staining areas were widely distributed across the thickened media and subintimal space, suggesting remarkable extracellular matrix deposition (Figure 2D). Quantitative analysis confirmed that the percentage of Masson-positive area in the VH group was significantly higher than that in the NC group (Figure 2F).

To further characterize the extracellular matrix remodeling, we performed immunofluorescence staining for Collagen I (COL1), Collagen III (COL3), and Fibronectin (FN) in venous sections from both groups (Figure S1). Quantitative analysis showed that the fluorescence intensities of COL1 and COL3 were significantly increased in the VH group compared with the NC group (COL1: p<0.0001; COL3: p=0.0004), indicating enhanced deposition of fibrillar collagens in the remodeled venous wall. In contrast, FN staining showed no statistically significant difference between the two groups (p=0.6438). These results suggest that the observed ECM remodeling is characterized predominantly by increased collagen deposition rather than a uniform increase in all ECM components, which is consistent with the Masson’s trichrome staining and the transcriptomic enrichment of ECM-related pathways.

Taken together, the left external jugular vein has developed prominent structural remodeling under hemodynamic load at 4 weeks post-surgery, as characterized by luminal dilation, wall hypertrophy, and excessive collagen fiber deposition.

### 3.3. Functional Enrichment Analysis of Differentially Expressed Genes

We systematically analyzed the differential gene expression profiles of the left external jugular vein between the VH and NC groups.

**Figure 3:**
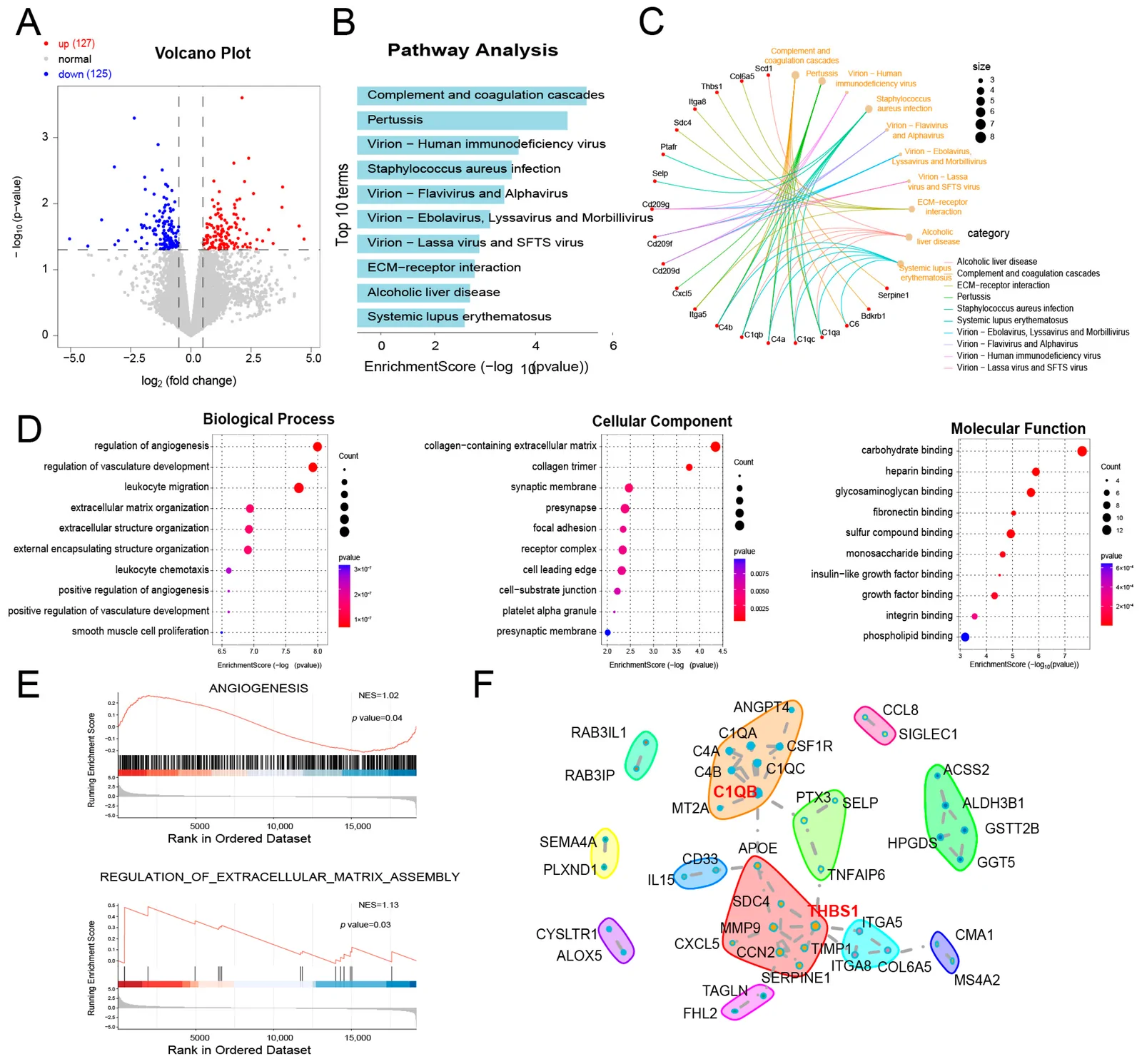
Transcriptomic analysis of venous tissues from the NC and VH groups. (**A**) Volcano plot of DEGs. Red, significantly upregulated genes; blue, significantly downregulated genes. (**B**) Hierarchical clustering heatmap of DEGs. (**C**) GO enrichment bubble plot covering BP and MF categories. (**D**) KEGG pathway enrichment bubble plot. (**E**) GSEA enrichment plots showing gene sets related to angiogenesis, ECM organization, and collagen fibril assembly. (**F**) PPI network of DEGs.

A total of 252 DEGs were identified, including 127 upregulated and 125 downregulated genes (Figure 3A). KEGG pathway enrichment analysis showed that DEGs were most significantly enriched in the complement and coagulation cascades pathway, and were also markedly enriched in ECM–receptor interaction and multiple viral infection-related pathways (Figure 3B,C).

GO enrichment analysis indicated that biological processes (BP) were significantly enriched in angiogenesis, extracellular matrix organization and collagen fibril organization; cellular components (CC) were mainly associated with collagen-containing extracellular matrix; and molecular functions (MF) were involved in extracellular matrix structural constituent and other terms (Figure 3D).

GSEA further confirmed that gene sets related to angiogenesis and extracellular matrix organization were significantly upregulated in the VH group (NES > 1, *p* < 0.05) (Figure 3E). PPI network analysis revealed that core hub nodes were mainly concentrated in the collagen family, integrins, and complement/coagulation-related proteins (Figure 3F).

### 3.4. Venous Hypertension Induces Downregulation of Smooth Muscle Contractile Markers

Previous studies on clinical samples of venous hypertensive remodeling have reported downregulated expression of VSMC contractile phenotypic markers α-SMA and SM22α, indicating a phenotypic switch of smooth muscle cells from a contractile to a synthetic/pro-remodeling phenotype [7,8]. In this study, we examined the protein levels of α-SMA and SM22α in the left external jugular vein of this novel model via immunofluorescence staining and Western blot, to verify whether VH induces similar contractile phenotypic alterations in this model.

**Figure 4:**
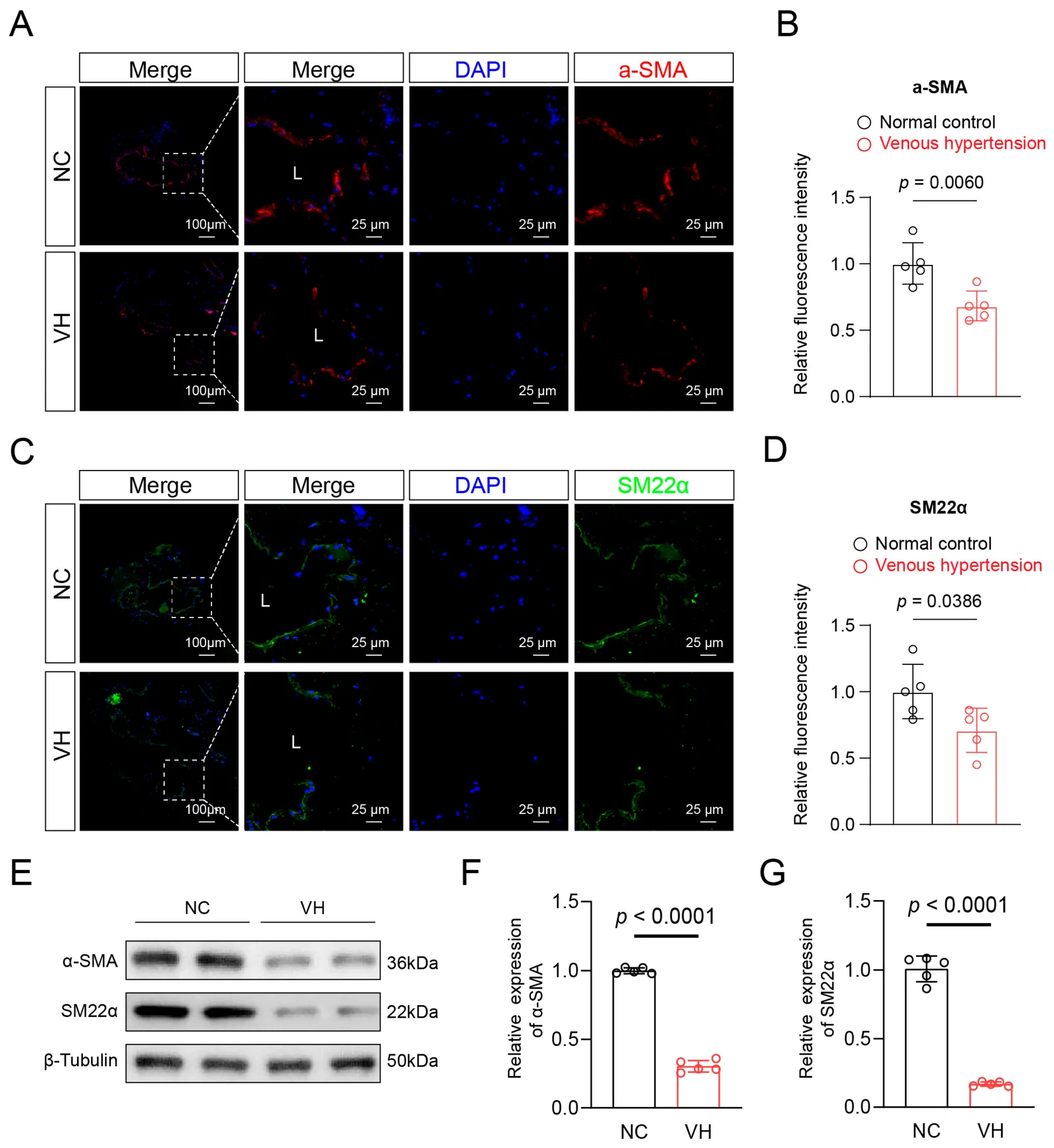
Immunofluorescence and Western blot analysis of smooth muscle contractile markers in venous tissues from NC and VH groups. (**A**) Immunofluorescence staining of α-SMA (red); merged images with DAPI (blue) nuclear counterstain; L indicates the vessel lumen. Scale bars: 100 μm (overview) and 25 μm (magnified insets). (**B**) Quantitative analysis of α-SMA immunofluorescence intensity (*p* = 0.0060, n = 5). (**C**) Immunofluorescence staining of SM22α (green); merged images with DAPI. (**D**) Quantitative analysis of SM22α immunofluorescence intensity (*p* = 0.0386, n = 5). (**E**) Representative Western blot bands of α-SMA (36 kDa), SM22α (22 kDa), and β-Tubulin (50 kDa, loading control). (**F**) Densitometric quantification of α-SMA relative protein expression (*p* < 0.0001, n = 5). (**G**) Densitometric quantification of SM22α relative protein expression (*p* < 0.0001, n = 5). Data are presented as mean ± SEM.

Immunofluorescence staining showed that both α-SMA and SM22α presented continuous, uniform, strong positive fluorescence signals in the venous wall of the NC group, mainly localized in the vascular media. In contrast, the fluorescence intensity of both markers was significantly reduced in the VH group, with sparse and discontinuous signal distribution (Figure 4A,C). Quantitative analysis confirmed that the mean relative fluorescence intensity of α-SMA and SM22α in the VH group was significantly lower than that in the NC group (Figure 4B,D). Western blot results showed a consistent trend with the immunofluorescence findings (Figure 4E–G).

These results indicate that VSMC contractile markers are also significantly downregulated in the novel VH remodeling model established in this study, which is consistent with the phenotypic alterations observed in varicose vein tissues in previous reports.

### 3.5. Venous Hypertension Induces Upregulation of Endothelial Adhesion Molecules

Previous studies on chronic venous disease and varicose veins have demonstrated that venous hypertension can trigger vascular endothelial cell activation, accompanied by upregulated expression of endothelial adhesion molecules ICAM-1 and VCAM-1 [9]. These findings suggest that endothelial inflammation and adhesion molecule upregulation are critical features of venous hypertension-related vascular lesions. However, whether similar alterations of endothelial adhesion molecules occur in the novel VH remodeling model remains unclear. Accordingly, we detected the protein expression of ICAM-1 in the left external jugular vein of this model via immunofluorescence staining and Western blot.

**Figure 5:**
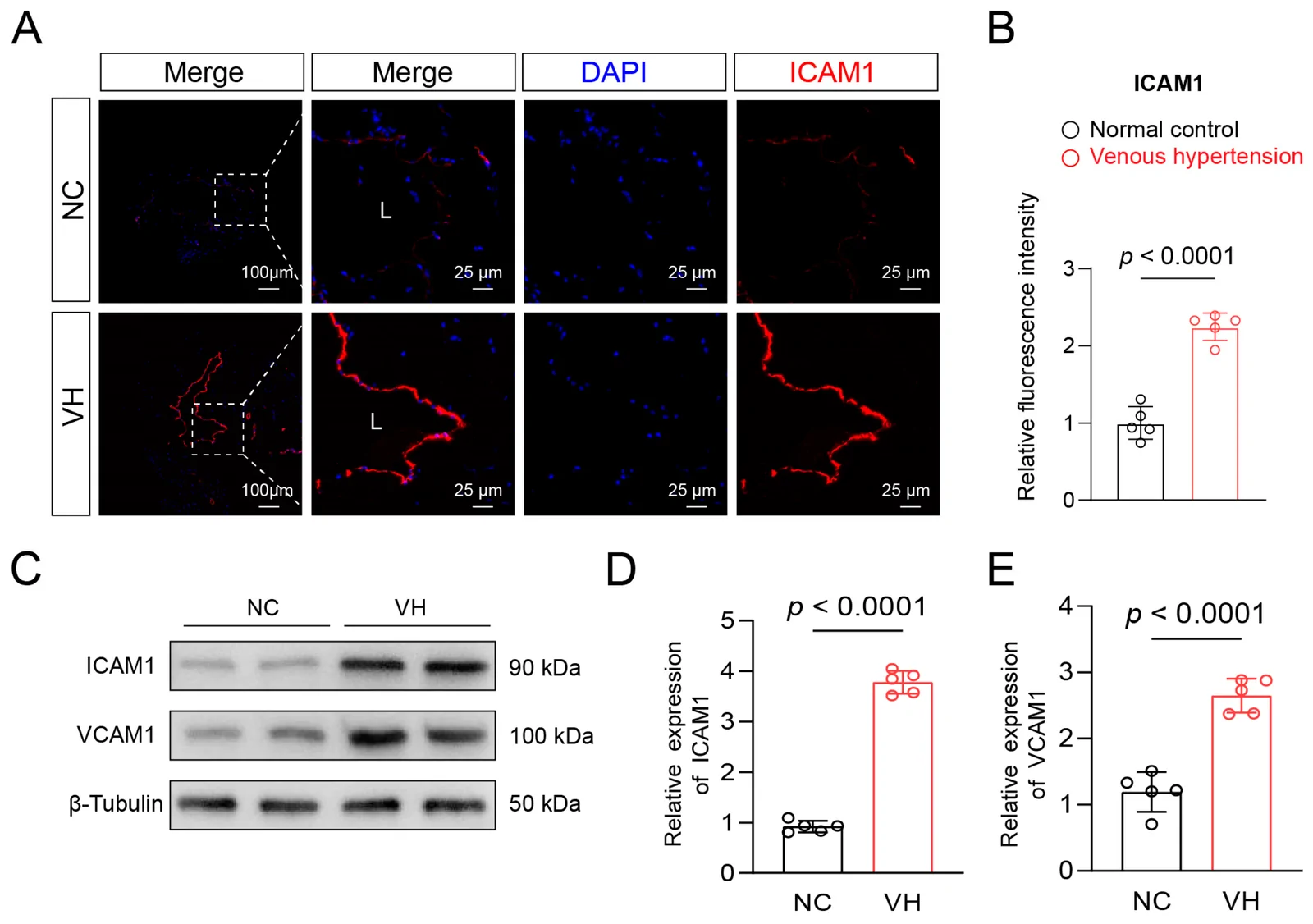
Immunofluorescence and Western blot analysis of endothelial adhesion molecules ICAM1 and VCAM1 in venous tissues from NC and VH groups. (**A**) Immunofluorescence staining of ICAM1 (red); merged images with DAPI (blue) nuclear counterstain; L indicates the vessel lumen. Scale bars: 100 μm (overview) and 25 μm (magnified insets). (**B**) Quantitative analysis of ICAM1 immunofluorescence intensity (*p* < 0.0001, n = 5). (**C**) Representative Western blot bands of ICAM1 (90 kDa), VCAM1 (100 kDa), and β-Tubulin (50 kDa, loading control). (**D**) Densitometric quantification of ICAM1 relative protein expression (*p* < 0.0001, n = 5). (**E**) Densitometric quantification of VCAM1 relative protein expression (*p* < 0.0001, n = 5).

Immunofluorescence staining showed that ICAM-1 exhibited weak positive signals in the venous wall of the NC group, while the fluorescence intensity of the adhesion molecule was significantly enhanced with a wider distribution in the VH group (Figure 5A,B). Western blot results were consistent with the immunofluorescence findings (Figure 5C–E). To confirm the endothelial localization of ICAM-1, we performed double immunofluorescence staining for ICAM-1 and the endothelial marker PECAM-1 (CD31) (Figure S2). The results showed clear spatial co-localization of ICAM-1 with PECAM-1-positive endothelial cells along the luminal surface of the venous wall. Moreover, ICAM-1 expression in the PECAM-1-positive endothelial region was markedly increased in the VH group compared with the Sham group, providing direct evidence supporting enhanced endothelial inflammatory activation following VH.

These results demonstrate that endothelial adhesion molecules ICAM-1 and VCAM-1 are also significantly upregulated in the novel VH remodeling model, which is consistent with the endothelial inflammatory alterations observed in chronic venous disease and varicose veins. This finding further validates that the novel model can effectively recapitulate the pathological features of VH-related endothelial inflammation.

## 4. Discussion

In the present study, we established a novel mouse VH remodeling model by selectively obstructing the unilateral cervical venous drainage system. At 4 weeks postoperatively, the compensatory left external jugular vein exhibited luminal dilation, wall thickening, and tortuous course. Histological examination revealed hyperplastic and disarranged medial smooth muscle cells along with increased collagen deposition, suggesting that this model was associated with structural remodeling resembling that induced by VH. These alterations are consistent with pathological features described in patients with varicose veins and chronic venous insufficiency, indicating that this model may partially reflect the pathological process of VH-induced remodeling in humans.

At the molecular level, the increased hemodynamic load was associated with downregulated expression of smooth muscle contractile phenotypic markers α-SMA and SM22α, suggesting a phenotypic switch of vascular smooth muscle cells from a contractile to a synthetic phenotype, consistent with findings in varicose vein tissues [10,11]. Meanwhile, the expression of endothelial adhesion molecules ICAM-1 and VCAM-1 was upregulated, suggesting that VH may activate vascular endothelial inflammatory responses, consistent with pathological alterations associated with VH [12]. Transcriptomic analysis revealed that differentially expressed genes were enriched in pathways such as complement and coagulation cascades and ECM–receptor interaction. GO and GSEA analyses indicated that biological processes including extracellular matrix organization, collagen fibril assembly, and angiogenesis were upregulated, in agreement with the fibrotic remodeling observed histologically. The PPI network suggested that collagen family members, integrins, and complement/coagulation-related proteins may constitute core regulatory nodes, providing a basis for subsequent mechanistic studies.

Of note, the enrichment of complement and coagulation cascade pathways in our transcriptomic data is consistent with emerging evidence from integrative bioinformatics analyses of venous disease. A transcriptomic meta-analysis demonstrated that innate immunity, complement activation, and classical hemostasis pathways are coordinately implicated in the pathogenesis of venous thrombosis and chronic venous disease at the RNA level [13]. Clinically, progressive chronic venous insufficiency is associated with elevated systemic markers of coagulation activation, including prothrombin fragments F1+2 and thrombin-antithrombin complexes, particularly in patients with active venous ulceration [14]. Furthermore, the complement and coagulation systems are recognized to engage in extensive crosstalk, whereby complement split products can induce tissue factor expression and platelet activation, thereby amplifying local pro-coagulant and inflammatory responses [15]. Nevertheless, the present study lacks direct protein-level validation of specific complement or coagulation components such as C1qb or fibrinogen (e.g., Western blot, ELISA, or targeted proteomics), because all venous tissue specimens from this cohort were consumed during histological, immunofluorescence, Western blot, and transcriptomic analyses. Future studies using targeted proteomics, ELISA, or Western blot are therefore needed to verify these transcriptomic observations at the protein level.

In the present study, we performed additional immunofluorescence staining for COL1, COL3, and FN to further characterize ECM remodeling, and conducted ICAM-1/PECAM-1 double staining to confirm endothelial localization of the inflammatory signal. The finding that FN was not significantly upregulated, despite increased COL1 and COL3 deposition, suggests that the ECM remodeling in this model is characterized by selective enhancement of fibrillar collagens rather than a global increase in all matrix components, which may reflect the specific pathological process induced by the hemodynamic load in this model. However, we acknowledge that VCAM-1/PECAM-1 double staining could not be reliably performed due to technical limitations. These technical and experimental constraints represent key priorities for future investigations.

Venous hypertension represents a key pathological driver in the initiation and progression of chronic venous disease. Sustained elevation of venous hydrostatic pressure compromises the mechanical integrity of the venous wall, promoting smooth muscle phenotypic switching, aberrant extracellular matrix deposition, and endothelial inflammatory cascades, which collectively contribute to luminal dilation, fibrotic wall thickening, and, in severe cases, venous ulceration [16,17]. Currently available animal models for investigating venous hypertension each have inherent limitations. Rat femoral arteriovenous fistula models produce tortuous dilation resembling human varicose veins; however, they require complex microsurgical anastomosis and carry a substantial risk of fistula failure or thrombosis [18]. Mouse hindlimb vein ligation models are technically straightforward, yet rapid collateral compensation often results in unstable pressure elevation [19]. Venous stenosis models more closely mimic chronic outflow obstruction but require several months to develop stable lesions. The mouse auricular vein ligation model allows dynamic observation of vascular tortuosity; nevertheless, the extremely small vessel caliber renders subsequent molecular or omics analyses impractical [20,21].

In comparison, the unilateral jugular vein transection model established in this study possesses several practical advantages over existing animal models for venous remodeling research. First, the surgical procedure is technically simple and easy to implement. Second, morphological alterations of the contralateral vein can be obviously observed within four weeks after intervention. Third, the vessel exhibits a sufficient caliber to support comprehensive histological, protein-level, and transcriptomic analyses. However, several limitations of this model still warrant careful consideration.

First, only a single time point at 4 weeks postoperatively was evaluated. This time point was selected based on preliminary experiments demonstrating significant vascular remodeling at this stage without excessive mortality, consistent with previous murine venous remodeling studies. Future studies incorporating additional time points (e.g., 1, 2, and 8 weeks) are warranted to dissect the temporal evolution of pathological changes. Second, direct intraluminal pressure of the compensatory vein was not measured. While this is a limitation, the observed phenotypes—including luminal dilation, wall thickening, downregulation of VSMC contractile markers, and upregulation of endothelial adhesion molecules—are well-established consequences of sustained VH. Future work will employ intravascular catheterization to quantify the pressure gradient directly. Third, this model simulates local high load of cervical veins, which differs from lower-extremity venous diseases in anatomical location and hemodynamic characteristics; thus, caution is warranted when extrapolating findings to clinical lower-extremity CVD.

## 5. Conclusions

In this study, we developed a novel mouse VH remodeling model via unilateral internal and external jugular vein ligation and transection. The model features a straightforward surgical procedure and displays characteristic pathological changes associated with VH. Reduced expression of smooth muscle contractile markers at both mRNA and protein levels, together with abnormal extracellular matrix deposition, collectively contribute to vascular wall remodeling in this setting. We expect that this model will provide a practical experimental platform for future investigations into the underlying mechanisms and potential therapeutic targets of VH-related vascular remodeling.

## Data Availability

The raw transcriptome sequencing data and gene expression count matrix generated in this study have been deposited in the NCBI Sequence Read Archive (SRA) under accession number PRJNA1493823. All other raw data supporting the conclusions of this study are available from the corresponding author upon reasonable request.

## Supplementary Materials

The following supporting information can be downloaded at https://www.mdpi.com/article/10.3390/jcdd13090452/s1, File S1: Raw uncropped western blot gel images; Figure S1: Extracellular matrix remodeling in vessels under venous hypertension; Figure S2: Endothelial localization of pro-inflammatory ICAM-1 under venous hypertension. Table S1: Differences and 95% confidence intervals of differences for quantitative comparison in bar graphs.

## Abbreviations

The following abbreviations are used in this manuscript:

α-SMA	α-Smooth Muscle Actin
BP	Biological Process
CC	Cellular Component
C1qb	Complement C1q subcomponent subunit B
CVD	Chronic Venous Disease
DAPI	4′,6-diamidino-2-phenylindole
DEGs	Differentially expressed genes
ECM	Extracellular Matrix
GO	Gene Ontology
GSEA	Gene set enrichment analysis
HE	Hematoxylin–Eosin
ICAM-1	Intercellular cell adhesion molecule 1
KEGG	Kyoto Encyclopedia of Genes and Genomes
MF	Molecular Function
NC	Normal Control
NES	Normalized enrichment score
PPI	Protein–protein interaction
RNA-seq	RNA Sequencing
SEM	Standard error of the mean
SM22α	Smooth Muscle 22 Alpha
VCAM-1	Vascular cell adhesion molecule 1
VH	Venous Hypertension
VSMC	Vascular smooth muscle cell
WB	Western Blot
