# Supplementary material for: A Novel Mouse Model for Venous High-Load Remodeling Induced by Unilateral Jugular Vein Transection
Source: J Cardiovasc Dev Dis. 2026 Sep 10;13(9):452. doi: 10.3390/jcdd13090452 (PMC13607619; doi:10.3390/jcdd13090452)
Supplement: Supplementary file 1 [file jcdd-13-00452-s001.zip › jcdd-4480661_Supplementary_Material.pdf]

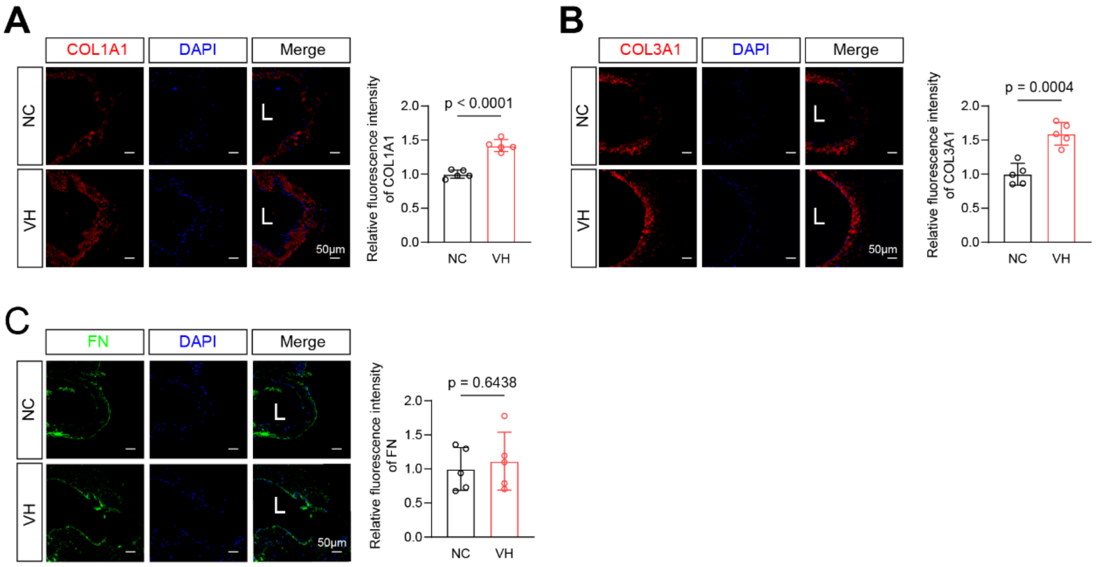

Figure S1. Extracellular matrix remodeling in vessels under venous hypertension. (A-B) Immunofluorescence staining and quantification showing significantly increased expression of Collagen I (COL1A1) and Collagen III (COL3A1) in venous-hypertensive (VH) vessels compared with normal control (NC). Red, COL1A1/COL3A1; blue, DAPI-labeled nuclei. (C) Fibronectin (FN) immunofluorescence and quantitative analysis. No significant inter-group difference in FN fluorescence intensity was observed. L, vessel lumen. Scale bar, 50 μm. (COL1: $p < 0.0001$; COL3: $p < 0.0004$; FN: $p = 0.6438$, $n = 5$ per group).

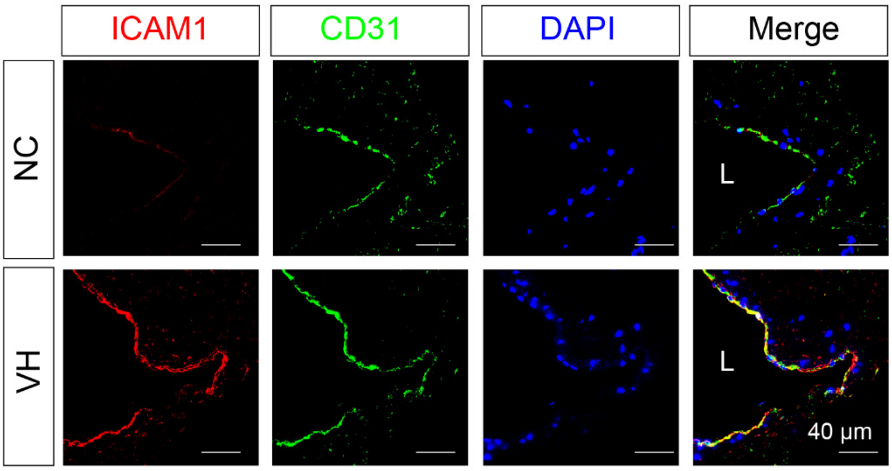

Figure S2. Endothelial localization of pro-inflammatory ICAM-1 under venous hypertension. Representative double-immunofluorescence images showing co-localization of ICAM-1 (red) with endothelial marker CD31 (green) in normal control (NC) and venous hypertension (VH) mouse veins. DAPI (blue) labels cell nuclei. Increased ICAM-1 signal was observed predominantly in CD31-positive endothelial layer in VH group. L, vessel lumen. Scale bar, 40 μm.
