# Supplementary material for: A Novel Mouse Model for Venous High-Load Remodeling Induced by Unilateral Jugular Vein Transection
Source: J Cardiovasc Dev Dis. 2026 Sep 10;13(9):452. doi: 10.3390/jcdd13090452 (PMC13607619; doi:10.3390/jcdd13090452)

Full unedited gel for Figure 4E α-SMA

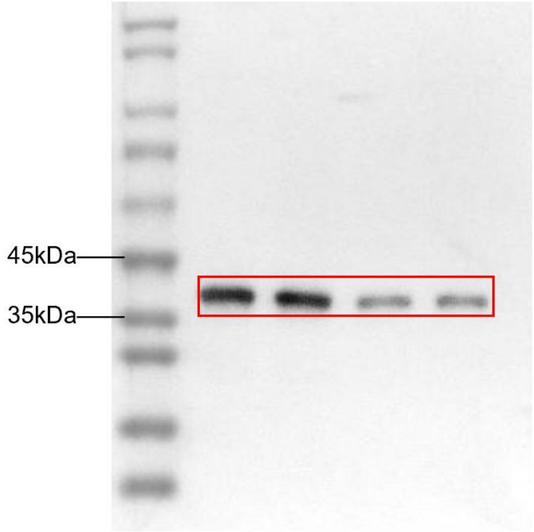

Full unedited gel for Figure 4E SM22α

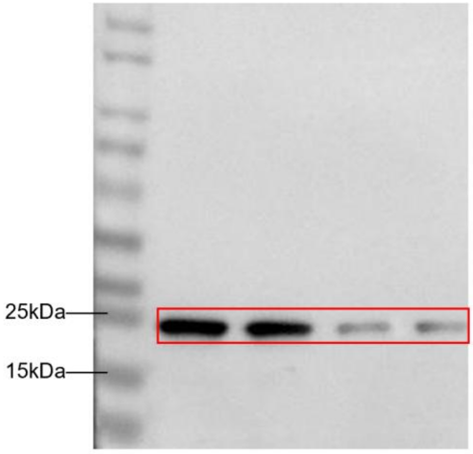

Full unedited gel for Figure 4E β-Tubulin

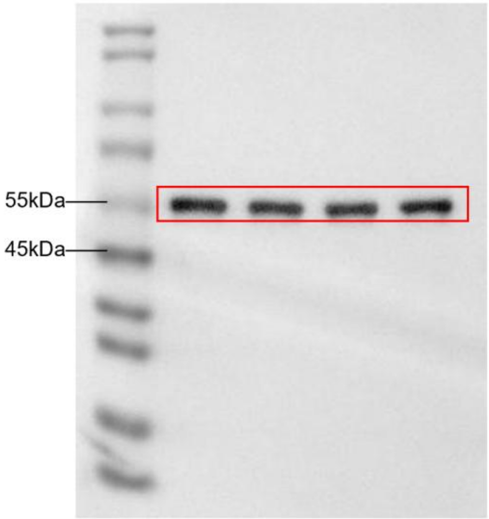

Full unedited gel for Figure 5C ICAM1

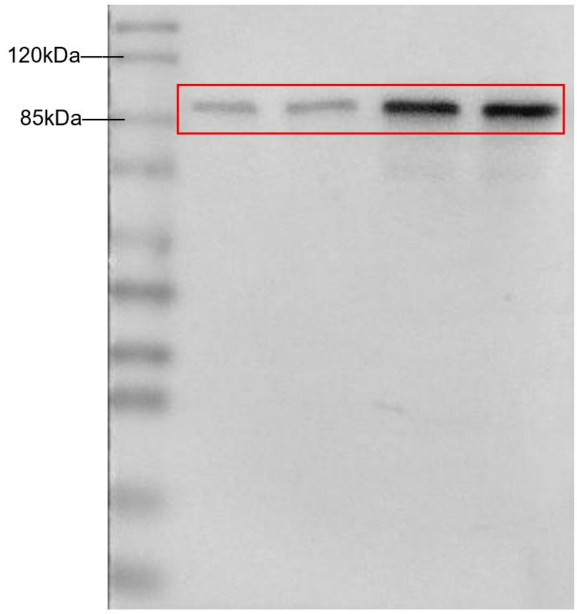

Full unedited gel for Figure 5C VCAM1

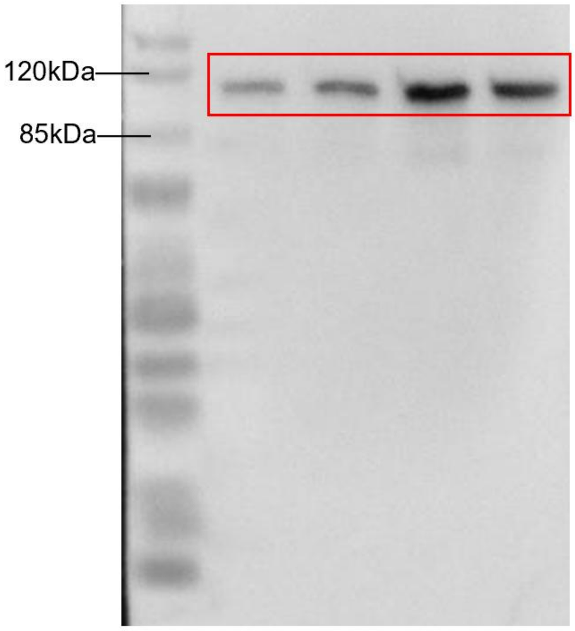

Full unedited gel for Figure 5C β-Tubulin

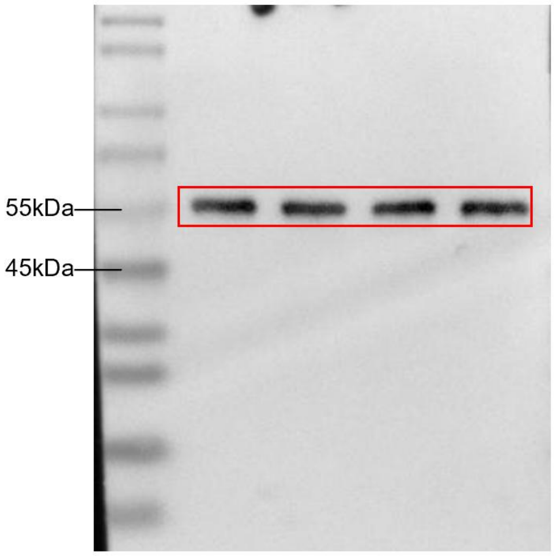

Supplement: Supplementary file 1 [file jcdd-13-00452-s001.zip › Supplementary_WB_Raw_Gel_Images.pdf]
